# Deterministic evolution and stringent selection during preneoplasia

**DOI:** 10.1038/s41586-023-06102-8

**Published:** 2023-05-31

**Authors:** Kasper Karlsson, Moritz J. Przybilla, Eran Kotler, Aziz Khan, Hang Xu, Kremena Karagyozova, Alexandra Sockell, Wing H. Wong, Katherine Liu, Amanda Mah, Yuan-Hung Lo, Bingxin Lu, Kathleen E. Houlahan, Zhicheng Ma, Carlos J. Suarez, Chris P. Barnes, Calvin J. Kuo, Christina Curtis

**Affiliations:** 1 Department of Medicine, Stanford University School of Medicine, Stanford, CA USA; 2 Department of Genetics, Stanford University School of Medicine, Stanford, CA USA; 3 Stanford Cancer Institute, Stanford University School of Medicine, Stanford, CA USA; 4 Science for Life Laboratory and Department of Oncology-Pathology, Karolinska Institutet, Stockholm, Sweden; 5 Department of Biology, Stanford University School of Medicine, Stanford, CA USA; 6 Department of Cell and Developmental Biology, University College London, London, UK; 7 Department of Pathology, Stanford University School of Medicine, Stanford, CA USA; 8 Department of Biomedical Data Science, Stanford University School of Medicine, Stanford, CA USA; 9 Chan Zuckerberg Biohub - San Francisco, San Francisco, CA USA; 10 Present Address: Wellcome Sanger Institute & University of Cambridge, Hinxton, UK

**Keywords:** Cancer genomics, Data integration

## Abstract

The earliest events during human tumour initiation, although poorly characterized, may hold clues to malignancy detection and prevention^1^. Here we model occult preneoplasia by biallelic inactivation of *TP53*, a common early event in gastric cancer, in human gastric organoids. Causal relationships between this initiating genetic lesion and resulting phenotypes were established using experimental evolution in multiple clonally derived cultures over 2 years. *TP53* loss elicited progressive aneuploidy, including copy number alterations and structural variants prevalent in gastric cancers, with evident preferred orders. Longitudinal single-cell sequencing of *TP53-*deficient gastric organoids similarly indicates progression towards malignant transcriptional programmes. Moreover, high-throughput lineage tracing with expressed cellular barcodes demonstrates reproducible dynamics whereby initially rare subclones with shared transcriptional programmes repeatedly attain clonal dominance. This powerful platform for experimental evolution exposes stringent selection, clonal interference and a marked degree of phenotypic convergence in premalignant epithelial organoids. These data imply predictability in the earliest stages of tumorigenesis and show evolutionary constraints and barriers to malignant transformation, with implications for earlier detection and interception of aggressive, genome-instable tumours.

## Abstract (web summary)

We model occult preneoplasia by biallelic inactivation of *TP53*, a common early event in gastric cancer, in human gastric organoids, the results implying predictability in the earliest stages of tumorigenesis.

## Main

In rapidly adapting asexual populations, including microorganisms and tumours, multiple mutant lineages often compete for dominance^2^. These complex dynamics determine the outcomes of evolutionary adaptation but are difficult to observe in vivo. Experimental evolution has yielded fundamental insights into clonal dynamics in microorganisms, enabling characterization of mutant clones and their fitness benefits^3,4^. The same forces of mutation and selection fuel clonal expansions in somatic cells during ageing, contributing to malignancy, but their dynamics are poorly understood^5–7^.

Cancers arise from a mutated cell that undergoes premalignant clonal expansion while accruing additional mutations. These mutations can spread in phenotypically normal tissues before apparent morphological changes, with aneuploidy and driver mutations preceding cancer diagnosis by years^5,8,9^. Identification of the causes of, and barriers to, malignant transformation requires characterization of the molecular phenotypes that precede this event in a tissue-specific manner. However, repeated sampling of healthy or preneoplastic tissue is impractical and thus evolutionary dynamics have been inferred from sequencing data^5,6,10^. For example, we inferred stringent subclonal selection in premalignant Barrett’s oesophagus, whereas matched adenocarcinomas largely exhibited neutral evolution^6^, presumably due to rapid growth after transformation and diminishing returns epistasis^11^. Despite these insights, the order of somatic alterations and patterns of clonal expansion that precede transformation are obscured in established cancers^5,12^, necessitating new approaches to empirically measure premalignant evolution.

Gastric cancer (GC), the fourth-leading cause of cancer mortality worldwide, lacks routine screening albeit its long lead times contributing to late diagnoses, poor prognosis and limited treatment options^13,14^. Therefore, it is crucial to identify the molecular determinants of GC and its non-obligate precursor, intestinal metaplasia, which is poorly characterized compared with precursor lesions in the adjacent oesophagus (Barrett’s oesophagus)^15–17^. Although the utility of forward-genetic and GC organoids as preclinical models has been established^18–21^, in the former, combinatorial hits were engineered to bypass nascent progression and accelerate transformation^19,21^.

Here we model tumorigenesis from the ‘bottom up’ using CRISPR–Cas9-engineered human gastric organoids (HGOs) to identify causal relationships between initiation of genetic insults and resultant genotypes and phenotypes. Because *TP53* inactivation is a common early event preceding numerical and structural chromosomal abnormalities (aneuploidy) in chromosomal instable (CIN) GC^19,22,23^, we use non-malignant HGOs as a tabula rasa to study preneoplasia induced by *TP53* deficiency over a 2-year time span. HGOs are ideal for this task because they recapitulate the cellular attributes of in vivo models, including three-dimensional tissue structure, multilineage differentiation and disease pathology^21^.

Whereas *TP53* is altered in over 70% of CIN GCs^22,23^, its ability to elicit aneuploidy, a hallmark of most solid cancers, has been controversial and appears tissue dependent^24–27^. Moreover, the extent to which specific copy number alterations (CNAs) are selectively advantageous, and their tumorigenic impact is largely unknown^28,29^. We chart genotype-to-phenotype maps of gastric preneoplasia following *TP53* inactivation in multiple HGO cultures and demonstrate that these models recapitulate genomic hallmarks of gastro-oesophageal tumorigenesis, including the multi-hit, temporal and repeated acquisition of CNAs and structural variants (SVs), accompanied by progression towards malignant transcriptional states. Prospective lineage tracing with linked single-cell expression profiles delineates early clonal dynamics, showing extensive clonal interference, stringent selection and rapid adaptation, underpinned by temporal genomic contingencies and phenotypic convergence. Our findings highlight the power of experimental evolution in human organoids to investigate occult preneoplastic processes and the repeatability of somatic evolution.

## *TP53*^–/–^ induces CNAs in defined orders

**Fig. 1:**
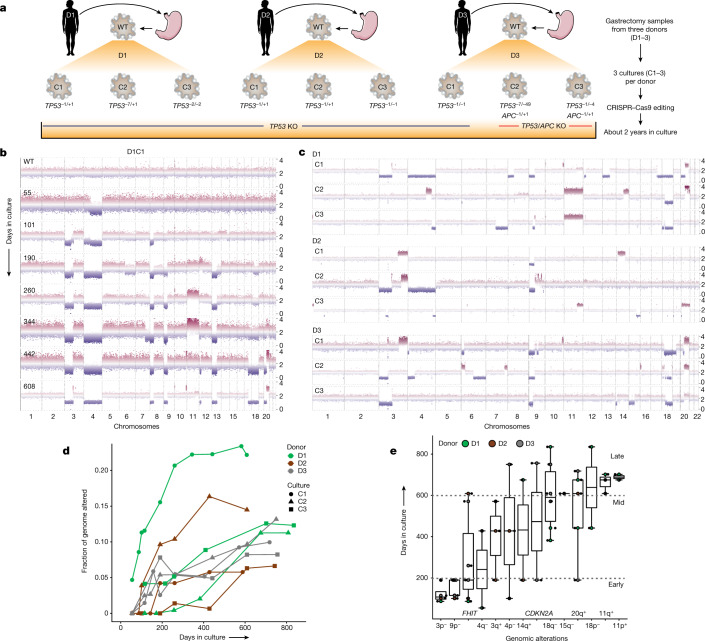
*TP53* deficiency in HGOs induces aneuploidy and GC-associated CNAs along a defined temporal order. **a**, Schematic overview of HGO establishment and generation of *TP53*^*–/–*^, *TP53*^*–/–*^ and *APC*^*–/–*^ cultures via CRISPR–Cas9 editing (Methods). **b**, Genome-wide CNA profiles of D1C1 at multiple time points assessed by sWGS. Normalized read counts across 50 kb genomic windows for each time point. **c**, CNA profiles for the nine organoid cultures sampled between days 588 and 835. **d**, FGA over time for each culture. **e**, Time of appearance (days in culture) of persistent arm-level CNAs (or alterations in *FHIT* and *CDKN2A*) in *TP53*/*APC*-deficient organoids (alterations that became extinct were not considered). The prevalence of these alterations in GC is summarized in Extended Data Fig. 2a,b. Boxes show interquartile range (IQR), centre lines represent the median and whiskers extend by 1.5× IQR. Sample size per aberration (*n*): 3p^–^ (4), 9p^–^ (7), *FHIT* (7), 4q^–^ (2), 3q^+^ (3), 4p^–^ (3), 14q^+^ (2), *CDKN2A* (2), 18q^–^ (6), 15q^–^ (2), 20q^+^ (5), 18p^–^ (2), 11q^+^ (3), 11p^+^ (2). KO, knockout. Image of stomach in **a** is from Servier Medical Art, CC BY 3.0. Source Data

**Extended Data Fig. 1:**
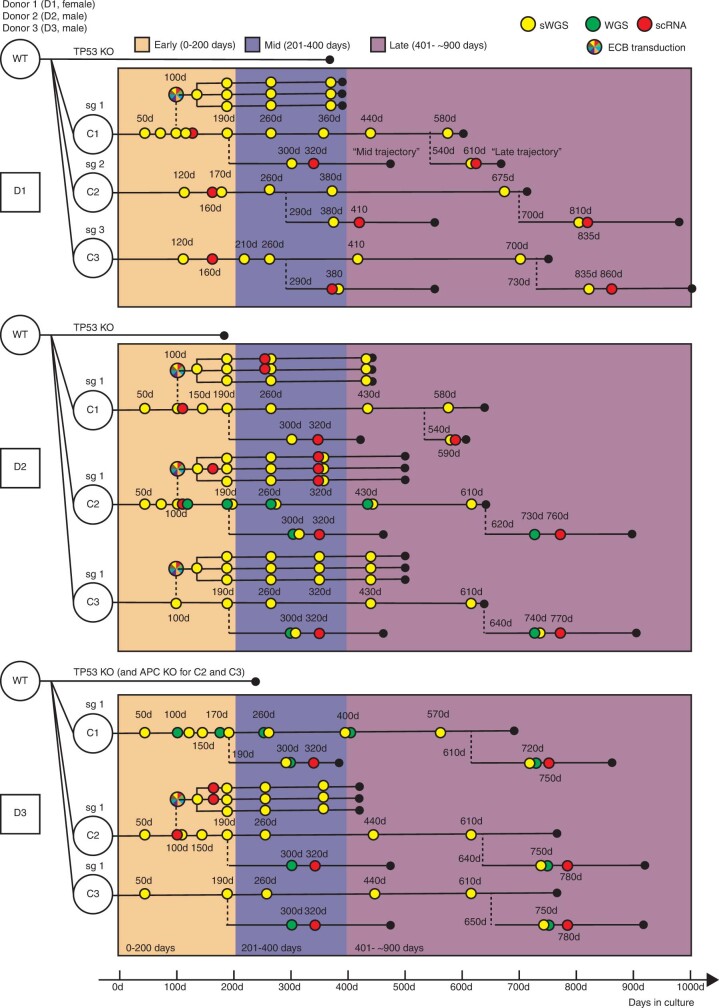
Schematic overview of gastric organoid cultures, assays and sequencing time points. Organoids were established from three donors (abbreviated D) as wild-type (WT) cultures. For each donor (D1-D3), three independent CRISPR/Cas9 edited *TP53*^*−/−*^ or *TP53*^*−/−*^*, APC*^*−/−*^ cultures (abbreviated C) were established (indicated by sg 1-3) and referred to as C1-C3 (Methods). The WT and genome edited cultures were evolved under defined conditions for over two years. Sequencing was performed across the experimental time course at defined intervals: *Early* (~0–200 days), *Mid* (~200–400 days), *Late* (~400–900 days). Each original culture was thawed (indicated by dashed lines) at an *Early/Mid* (190–290 days) and *Late* (540–730 days) time point for additional replication and comparisons. The thawed samples were treated with normocin to eliminate mycoplasma (Methods). All cultures were subject to shallow WGS (sWGS). A subset of cultures underwent deeper WGS and/or single cell RNA (scRNA)-sequencing at select time points. In addition to these non-barcoded cultures, representative *TP53*^*−/−*^ HGO cultures from each donor were selected for prospective lineage tracing via transduction of a lentiviral expressed cellular barcode (ECB), as indicated by the multi-coloured circle in the legend. These ECB cultures were similarly subject to sWGS and scRNA-seq. Broad time intervals are indicated as in the legend, while days in culture are provided for individual cultures. Note that scRNA for D1C3 “Mid trajectory” was sampled at day 413.

**Extended Data Fig. 2:**
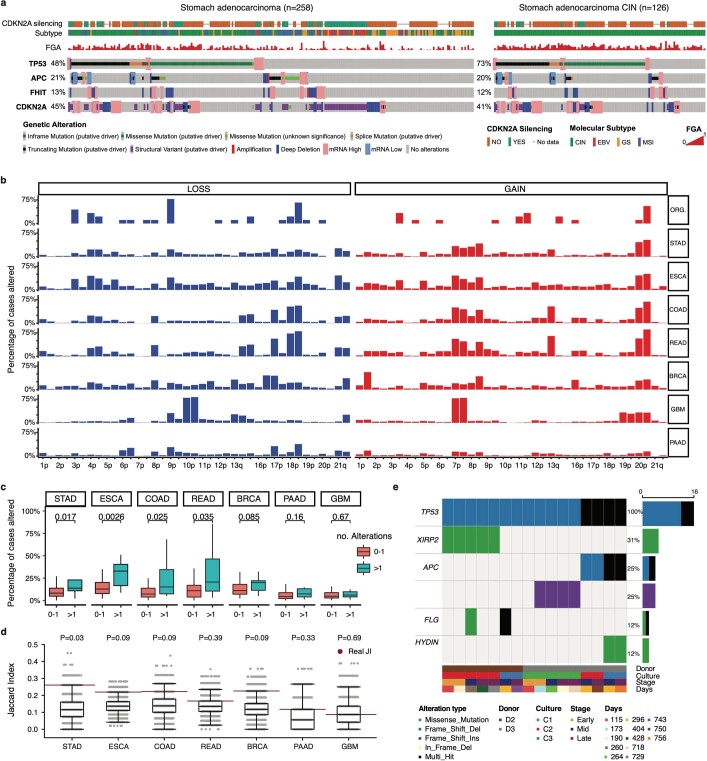
Recurrent copy number aberrations in *TP53*-deficient gastric organoids are enriched in gastric and esophageal cancers. **a**, Prevalence of somatic alterations and fraction genome altered in gastric cancer (stomach adenocarcinoma, STAD) from TCGA in all subgroups versus the CIN subgroup. Data derive from the cBioPortal. **b**, Frequency of chromosome arm alterations in *TP53*^−/−^ HGOs (ORG) at late time points (days 588 to 835, as in Fig. 1c) relative to other tumour types: Stomach Adenocarcinoma (STAD), Esophageal carcinoma (ESCA), Colorectal Adenocarcinoma (COAD), Rectum Adenocarcinoma (READ), Breast invasive carcinoma (BRCA), Glioblastoma Multiforme (GBM), Pancreatic Adenocarcinoma (PAAD). TCGA data were obtained from Firehose (http://gdac.broadinstitute.org/#). **c**, Enrichment of chromosome arm-level alterations in *TP53*^−/−^ gastric organoid cultures across cancer types. Boxes show inter-quartile range (IQR), center lines represent the median, whiskers extend by 1.5 × IQR. Arm-level CNAs altered in two or more *TP53*^−/−^ gastric organoid cultures (n = 12 alterations) were more frequently altered than alterations present in 1 or fewer cultures (n = 66 alterations) in both STAD and ESCA (p-value shown, two-sided Wilcoxon rank sum test). **d**, The Jaccard Index (JI) was calculated by comparing CNAs that occurred in more than 1 chromosome arm in organoid cultures with CNAs occurring in 15% or more cases in a given tumour type. Permutation tests were performed to determine if the JI score was higher than expected by chance (i.e. one-sided test). For each tumour type, organoid CNA labels were randomly sampled (n = 10,000) from all possible chromosome arm-level events, and the JI was calculated. The empirical p-value was calculated as: P = 1-(sum(real JI > null JIs)/number of null JIs). Boxplot represents median, 0.25 and 0.75 quantiles with whiskers at 1.5 × IQR and includes the nominal p-values. **e**, Oncoplot shows alterations that occurred in two or more samples for genes commonly (>10% of cases) altered in gastro-esophageal cancer. Source Data

To model tumour initiation in CIN GC, we established HGOs from non-malignant tissue from three human donors undergoing gastrectomy and introduced biallelic *TP53* frameshift mutations via CRISPR–Cas9, resulting in an inactive gene product (Fig. 1a, Extended Data Fig. 1, Supplementary Figs. 1–3, Supplementary Tables 1 and 2 and Methods). From each donor (D1–3), three independent, clonally derived *TP53*^*–/–*^ cultures (C1–3) were established, yielding nine cultures for long-term propagation, five of which were each split into three replicates (R1–3) for cellular barcoding studies (*n* = 24 cultures). Another ‘hit’ in the *APC* tumour suppressor, a Wnt pathway negative regulator altered in 20% of CIN GC (Extended Data Fig. 2a), was concurrently engineered in C2 and C3 from D3 (referred to as D3C2 and D3C3, respectively; Supplementary Figs. 1 and 3) to examine the evolutionary consequence of dual tumour suppressor inactivation. The clonal status of CRISPR-edited sites was verified via Sanger sequencing and confirmed by whole-genome sequencing (WGS) at multiple time points (Supplementary Figs. 1–3). Throughout, we refer to time as days after *TP53* deficiency was engineered and we group *TP53*^–/–^ and *TP53*^*–/–*^ and *APC*^*–/–*^ cultures unless otherwise specified.

We first asked whether *TP53* deficiency elicits aneuploidy, measured as numeric and/or structural chromosomal abnormalities (genome instability). To investigate CNAs we sequenced the nine cultures (shallow whole-genome sequencing, sWGS; median 0.2× coverage) at up to 11 time points, spanning early (0–200 days), mid (around 200–400 days) and late (around 400–900 days) intervals (Extended Data Fig. 1 and Supplementary Table 3). *TP53-*deficient organoids progressively acquired CNAs, first accruing chromosome arm-level losses followed by copy number gains (Fig. 1b). By contrast, wild-type (WT) gastric organoids remained genomically stable in the long term (13–26 passages; Supplementary Figs. 4–6). Cultures from the same donor exhibited variable CNA patterns, suggesting that genetic background does not wholly constrain subsequent alterations (Fig. 1c and Supplementary Figs. 4–6). Despite this variability, CNAs prevalent in the TCGA GC cohort were recurrently altered in *TP53*^*–/–*^ cultures, including loss of chromosome (chr) 3p, 9p and 18q and gain of 20q (Extended Data Fig. 2)^22^. Additionally, arm-level CNAs present in two or more *TP53*^–/–^ HGOs were enriched in gastric and oesophageal cancers but not in other tumour types (*P* < 0.05, two-sided Wilcoxon rank-sum test; Extended Data Fig. 2c). Thus, recurrent tissue-specific CNAs accrue in *TP53*^*–/–*^ HGOs. During the experiment, because mycoplasma was detected in early-passage WT cultures and some derivative samples an antibiotic (normocin) was used to eliminate infections (Methods and Supplementary Fig. 7a–f). Accordingly replicate experiments were performed under mycoplasma-free conditions, demonstrating that mycoplasma infection is not associated with CNAs or other molecular features (Extended Data Fig. 1, Methods and Supplementary Figs. 7g and 8).

Across all cultures the fraction of genome altered (FGA), a measure of aneuploidy, increased over time at varying rates and plateaued around day 600 (Fig. 1d and Methods). For example, D1C1, which accrued early arm-level alterations, exhibited over 20% FGA by day 260 compared with a median FGA of about 5% across all cultures at similar time points. *TP53*^*–/–*^ and *TP53*^*–/–*^/*APC*^*–/–*^ cultures exhibited comparable FGA at final time points (average 11.3 and 10.7%, respectively), consistent with the expectation that *APC* loss does not fuel gastric cell aneuploidy. In several cultures FGA decreased over an interval due to clonal extinction (D3C3 day 190 versus day 442; D2C2 day 428 versus day 609) (Fig. 1d and Supplementary Figs. 5 and 6). As expected, FGA was lower in *TP53*^*–/–*^ HGOs than in CIN GCs (median FGA 34.5% in TCGA, according to cBioPortal).

Investigation of the temporal onset of arm-level and focal CNAs in *TP53*^*–/–*^ HGOs showed preferred orders (Fig. 1e and Supplementary Table 3). Specifically, loss of chr9p and chr3p repeatedly occurred (across donors and cultures) within 200 days but seldom later, suggesting a period during which these alterations were particularly advantageous. Chr9p deletion spans the *CDKN2A* tumour suppressor commonly altered in the CIN subgroup of gastric (roughly 41%; Extended Data Fig. 2a) and oesophageal (roughly 74%) adenocarcinomas, and co-occurs with *TP53* alterations^19,22^. Indeed, *CDKN2A* loss signals the initiation of Barrett’s oesophagus progression to dysplasia and oesophageal adenocarcinoma^30^ and GC premalignancy^19^. Deeper sequencing confirmed biallelic loss of *CDKN2A* via focal deletion (D3C1, D3C3) or truncating mutations in p16 (*INK4A*), along with heterozygous loss (D1C3) (Supplementary Fig. 9 and Supplementary Table 3). Similarly, deletion of the FHIT/FRA3B protein encoded on chr3p commonly occurred early in *TP53*^*–/–*^ HGOs (median 190 days) (Fig. 1e, Supplementary Fig. 10 and Supplementary Table 3). A genome caretaker, *FHIT*, is lost early during tumour progression leading to deoxythymidine triphosphate depletion, replication stress and DNA breaks^31^. Notably, 12% of CIN GCs harbour *FHIT* alterations (Extended Data Fig. 2a). Although *CDKN2A* and *FHIT* deletions are insufficient for malignant transformation^15,32^, their recurrent early loss during in vitro evolution and in GCs implies a role in tumour initiation. Additional GC-associated CNAs include loss of chr18q and gain of chr20q, which consistently occurred late (around 600 days). Such late alterations may reflect dynamic selective pressures from increased fitness or new evolutionary paths enabled by earlier alterations^4^. These data demonstrate that *TP53* loss facilitates aneuploidy in gastric cells and accrual of tissue-specific CNAs in a defined order.

## Selection and clonal interference

**Fig. 2:**
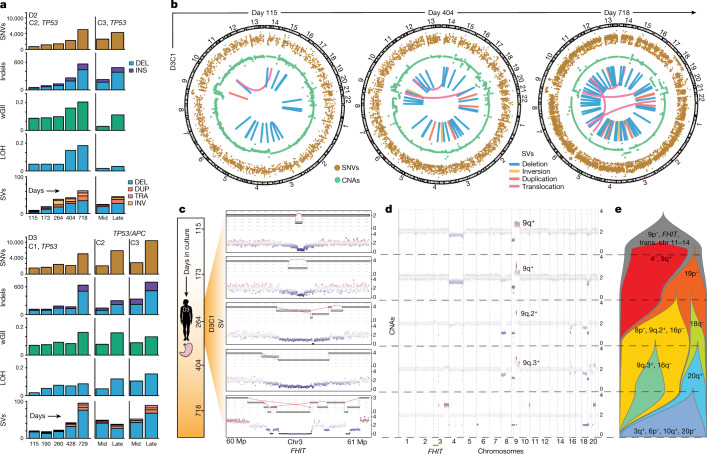
*TP53* deficiency elicits subclonal copy number evolution, SVs and clonal interference. **a**, Burden of different classes of somatic genomic alterations in *TP53*^*–/–*^ and *TP53*^*–/–*^, *APC*^*–/–*^ HGOs (relative to WT over time), as assessed by longitudinal WGS of individual cultures at the specified time points (mid, day 296; late, days 743–756). **b**, Circos plots for D3C1 illustrating increasing genomic instability and complexity over time. Classes of alterations shown include SNVs (adjusted variant allele frequencies), CNAs (log(*R*)) and SV consensus calls (Methods). **c**, Evolution of rigma-like SVs at the *FHIT* fragile site on chr3p. Zoomed-in view of a 1 Mb region in the *FHIT* locus. Top, reconstructed SVs; bottom, corresponding CNAs. **d**, Longitudinal CNA profiles for D3C1. **e**, Fishplot schematic for D3C1 illustrating subclonal CNA evolution, clonal interference and extinction. Subclone frequencies (*x* axis) were determined based on CNAs visualized in **d** (Methods). DEL, deletion; DUP, duplication; INV, inversion; TRA, translocation. Image of stomach in **c** is from Servier Medical Art, CC BY 3.0. Source Data

**Extended Data Fig. 3:**
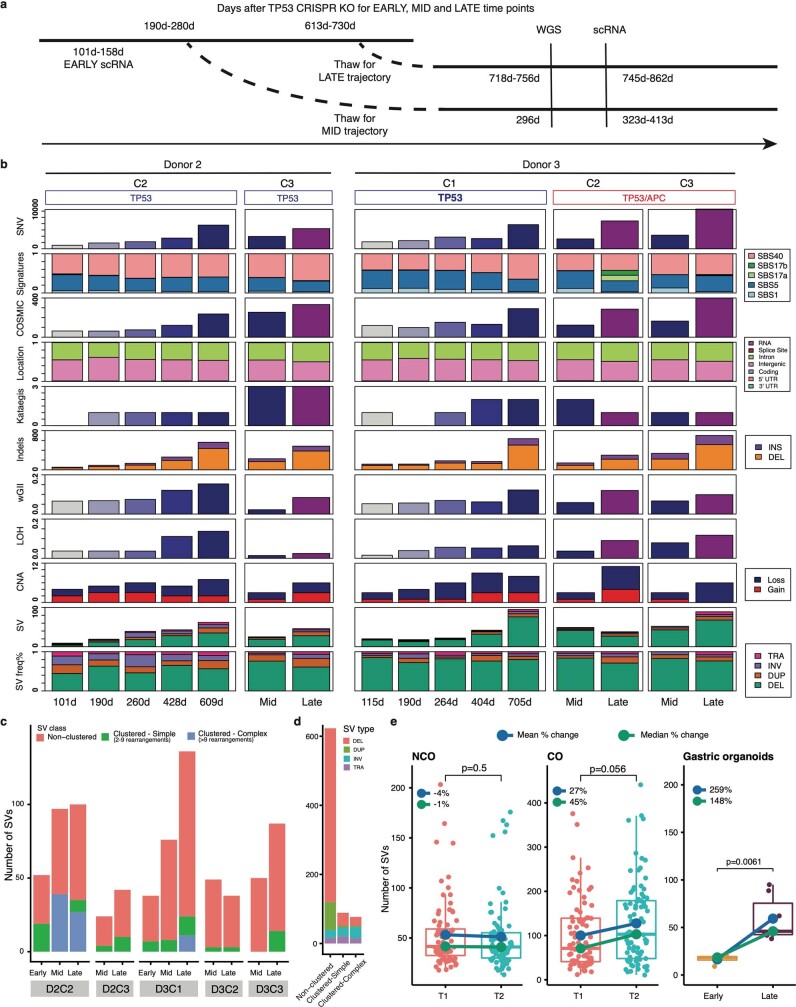
Longitudinal whole genome sequencing (WGS) of *TP53*^*−/−*^ gastric organoids . **a**, Overview of WGS and scRNA-seq time points for Early, Mid and Late cultures. Time is indicated in days (d). **b**, Summary of genomic features as assessed by WGS of multiple time points for Donors 2 and 3, expanding upon Fig. 2a. *Mid* and *Late* time points correspond to day 296 and 705–754, respectively. **c**, Distribution of non-clustered SVs, simple SVs (2–9 rearrangements) and complex SVs (10 or more rearrangements) defined using ClusterSV (Methods). **d**, Distribution of SV types across the three classes of SVs (non-clustered, clustered-simple and clustered-complex) **e**, Boxplot comparing total SV burden at the time of endoscopy (T1 and T2) for four Barrett’s esophagus biopsies per patient with cancer outcome (CO, n = 160) or noncancer outcome (NCO; n = 160) from the Paulson et al. cohort relative to the total SV burden between early (n = 4) and late (n = 9) timepoints in *TP53*^*−/−*^ and *TP53*^*−/−*^*, APC*^*−/−*^ HGOs (right). P-values were calculated using Wilcoxon rank sum test (two sided, unpaired). Boxes show IQR, center lines represent the median, whiskers extend by 1.5 × IQR.

We next sequenced (WGS, mean coverage 26×) five *TP53*^*–/–*^ cultures at multiple time points (Fig. 2a, Extended Data Fig. 3a and Supplementary Table 4). This confirmed biallelic *TP53* and *APC* inactivation at CRISPR target sites (Supplementary Fig. 2) and showed an increase in the weighted genome instability index (wGII), the fraction of genome with loss of heterozygosity (LOH), as well as focal deletions and amplifications during prolonged culture (Fig. 2a, Extended Data Fig. 3b and Supplementary Table 4). Single-nucleotide variants (SNVs) and SVs also increased over time (Fig. 2a, Extended Data Fig. 3b and Supplementary Table 4). At late time points the SNV burden was higher in *TP53*^*–/–*^/*AP*C^*–/–*^ (D3C2, D3C3) than in *TP53*^*−/–*^ HGOs. Few GC-associated genes were mutated across donors (Extended Data Fig. 2e).

Several regions of densely clustered mutations (hypermutation) were noted, including the *FHIT* fragile site in all sequenced cultures (Supplementary Figs. 11–13 and Supplementary Table 3). WT cultures exhibited simple focal *FHIT* deletions at late passages, probably due to clonal expansion of an initially rare event, and suggestive of somatic mosaicism (Supplementary Figs. 11a,b and 12a,b). Single-base substitutions 1, 5 and 40, which are ubiquitous and implicated in ageing and cancer, were the most prevalent mutational signatures^33^. However, by the late time point D3C2 developed single-base substitution 17a/b (Extended Data Fig. 3b and Supplementary Table 4), which is prevalent in gastro-oesophageal carcinomas and progressive Barrett’s oesophagus lesions^32^.

All classes of alterations accumulated in evolved *TP53*^*–/–*^ HGOs but SVs were particularly notable, with non-clustered and simple clustered rearrangements dominating at early time points followed by complex clusters (ten or more rearrangements) involving deletions, inversions and translocations over time (Fig. 2a and Extended Data Fig. 3c,d). This is exemplified by D3C1, which accrued multiple interchromosomal rearrangements (Fig. 2b). Although such complex SVs are seldom reported in normal tissues, they are prevalent in progressive Barrett’s oesophagus^16^. SV burden increased markedly in *TP53*^*–/–*^ HGOs between early and late time points (median change of 148%), exceeding by over threefold the change in SV burden (45%) between endoscopies in patients with Barrett’s oesophagus harbouring biallelic *TP53* inactivation and who subsequently progressed to oesophageal adenocarcinoma (average 2.2 years, range 0.65–6.16 years)^16^. By contrast, Barrett’s oesophagus non-progressors (lacking *TP53* biallelic inactivation) had a low and stable SV burden between endoscopies (Extended Data Fig. 3e).

**Extended Data Fig. 4:**
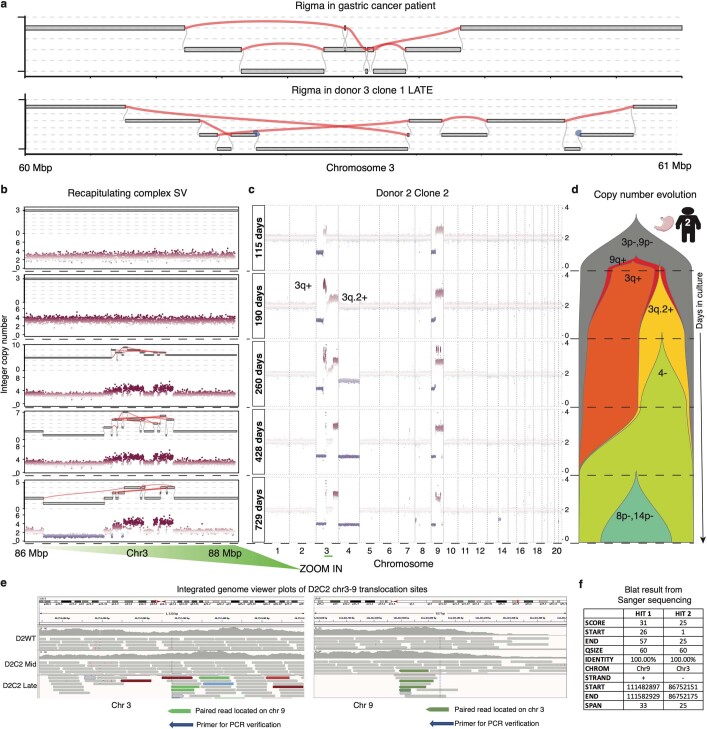
*TP53*-deficient gastric organoids recapitulate complex structural variants (SVs) observed in gastric cancers. **a**, Complex rigma-like SVs seen in gastric cancer (GC) patients such as pfg008 from the Wang et al. cohort (Methods) are similar to those in *TP53*^*−/−*^ HGOs including D3C1 (also shown in Fig. 2e). **b**, Zoomed-in view of a region on chromosome 3 which evolved complex SVs during *in vitro* culture. **c**, Corresponding CNA profiles based on longitudinal WGS of D2C2 at 5 timepoints spanning days 115 to 729 in culture. **d**, Fishplot for D2C2 depicting CNA evolution inferred from WGS. **e**, IGV plots indicate the translocation between chromosomes 3 and 9 for D2C2, corresponding to the complex SV in panel b. Primers used for PCR amplification, gel electrophoresis purification and Sanger sequencing as marked in the plots **f**, BLAT results from Sanger sequencing of the PCR primers demonstrating partial alignment to chr3 and chr9. Image of stomach in **d** is from Servier Medical Art, CC BY 3.0. Source Data

The *FHIT* locus frequently harboured complex SVs, including deletion chasms at fragile sites (rigma), as reported in GC and Barrett’s oesophagus^34^ (Fig. 2c, Extended Data Fig. 4a and Methods). We traced the genesis of rearrangements at the *FHIT* in D3C1, starting from a small deletion at day 115 and culminating in rigma by day 264. The subclone harbouring this rearrangement was lost (Fig. 2d,e, yellow subclone) but a separate subclone (blue) with a distinct *FHIT* rigma emerged and persisted, suggesting convergent evolution. Thus, rearrangements with multiple junctions evolve over several generations, not as a single event as previously proposed^35^. Similar events evolved in other cultures, including a chr3 and chr9 translocation (Extended Data Fig. 4b–f and Supplementary Fig. 14a,b). Despite these rearrangements, overall genomic content remained diploid as confirmed by flow cytometry (Supplementary Fig. 14c and Supplementary Table 4).

Clonal competition and extinction were investigated by determination of subclonal populations from CNA profiles (via bulk WGS) across five time points for D3C1 and D2C2. By day 115, D3C1 had acquired numerous deletions (9p, *FHIT*) and several SVs, including a persistent chr11–chr14 translocation. Over 600 days, multiple CNA-defined subclones increased in frequency before extinction (Fig. 2d,e and Supplementary Table 5). For example, a chr4^–^, 9q^+^ subclone arose early but disappeared by day 264, outcompeted by a chr19p^–^ subclone that later acquired chr8p^–^, 9q.2^+^ and 16p^–^ alterations and remained dominant until day 404. This subclone was ultimately outcompeted by one with chr18q loss that acquired gain of chr20q, both recurrent late events in multiple cultures. Thus, some clones fix and achieve dominance whereas others reach substantial frequencies before going extinct, presumably due to clonal interference. Distinct CNA subclones coexisted for extended durations (around 140 days), suggesting comparable fitness (for example, chr8p^–^, 9q.2^+^, 16p^–^ and 18q^–^ subclones) and intermittent periods of clonal competition and stasis as seen in other cultures (Extended Data Fig. 4c,d). These data demonstrate stringent selection and pervasive clonal interference in premalignant epithelial populations.

## Transcriptional changes following *TP53*^–/–^

**Fig. 3:**
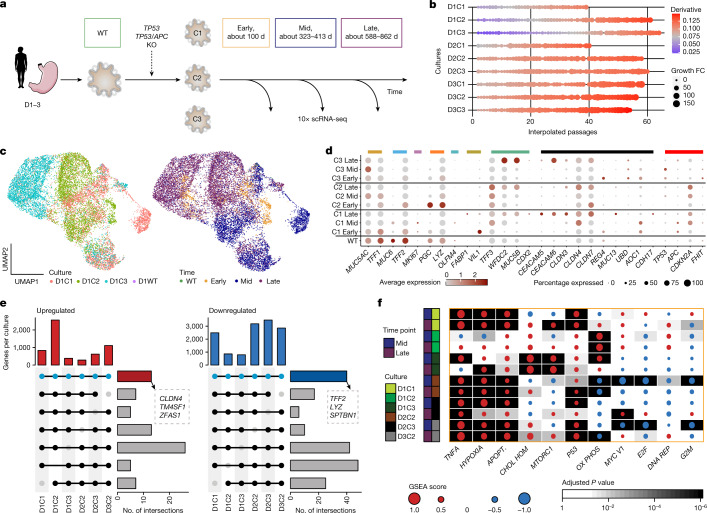
Transcriptional deregulation in *TP53-*deficient gastric organoids. **a**, Experimental overview of longitudinal scRNA-seq profiling of gastric organoid cultures. WT and replicate *TP53*^*–/–*^ HGOs were sampled at multiple time points (early, about 100 days, orange; mid, about 320 days, blue; late, about 770 days, purple) and subjected to scRNA-seq. **b**, Dot-plot depicting estimated growth curve derivatives and growth fold change (FC) from previous time points for each culture over time (interpolated passage number). **c**, Uniform manifold approximation and projection (UMAP) visualizations coloured according to culture (left) and time point (right) for D1, depicting 13,984 cells. **d**, Dot-plot depicting the expression of selected marker genes for individual cultures and time points. Coloured bars highlight (1) marker genes associated with normal gastric and intestinal cell types, (2) genes upregulated in gene expression profiling interactive analysis (GEPIA) of GC and (3) others of functional relevance. PMCs, *MUC5AC*, *TFF1*, dark yellow; GMCs, *MUC6*, *TFF2*, light blue; proliferative cells, *MKI67*, purple; neck-like cells, *PGC*, *LYZ*, orange; mucosal stem cells, *OLFM4*, turquoise; enterocytes, *FABP1*, *VIL1*, olive; goblet cells, *TFF3*, *WFDC2*, *MUC5B*, *CDX2*, green; GEPIA top 12 genes, *CEACAM5*, *CEACAM6*, *CLDN3*, *CLDN4*, *CLDN7*, *REG4*, *MUC3A*, *MUC13*, *PI3*, *UBD*, *AOC1*, *CDH17*, black; other, *TP53*, *APC*, *CDKN2A*, *FHIT*, red. **e**, UpSet plot representing shared differentially up- (left) and downregulated genes (right) across donors and cultures (*P* < 0.05, Bonferroni corrected two-sided Wilcoxon rank-sum test). **f**, GSEA heatmap for MsigDB Hallmark gene sets showing pathways most significantly altered for each culture (Kolmogorov–Smirnov statistic, Benjamini–Hochberg adjusted, two-sided). GSEA score is indicated (dot size) and coloured according to the directionality of expression profiles (up, red; down, blue). Image of stomach in **a** is from Servier Medical Art, CC BY 3.0. Source Data

**Extended Data Fig. 5:**
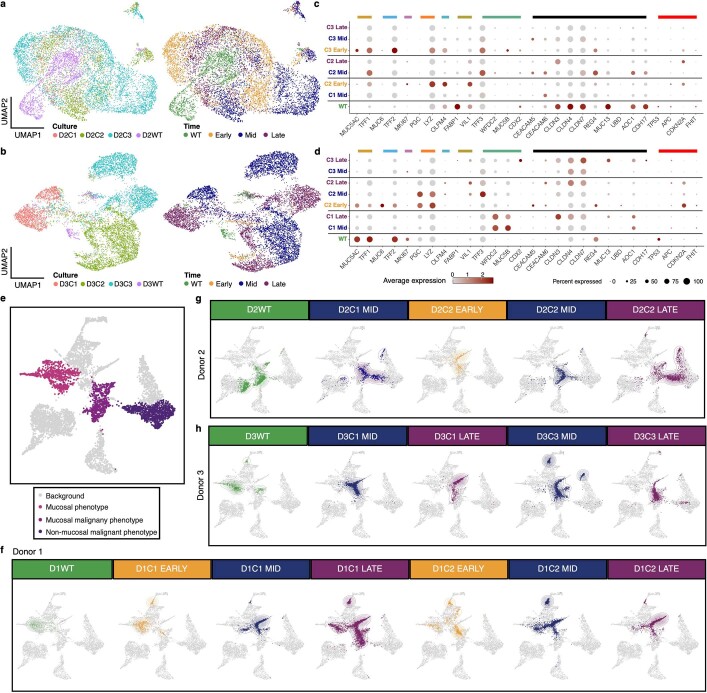
Latent Semantic Index (LSI) projection of gastric organoids onto gastric tissue dataset. **a–b**, UMAP visualizations coloured according to culture (left) and time point (right) for Donors 2 and 3 depicting 9,031 and 8,591 cells, respectively. **c–d**, Dotplot depicting the expression of selected marker genes for individual cultures and time points. Coloured bars highlight marker genes associated with normal gastric and intestinal cell types, genes up-regulated in the gene expression profiling interactive analysis (GEPIA) of GC, and others of functional relevance. Pit mucosal cells: *MUC5AC*, *TFF1* – dark yellow; Gland mucosal cells: *MUC6*, *TFF2* – light blue; Proliferative cells: *MKI67* – purple; Neck-like cells: *PGC*, *LYZ* – orange; Mucosal stem cells: *OLFM4* – turquoise; Enterocytes: *FABP1*, *VIL1* – olive; Goblet cells: *TFF3*, *WFDC2*, *MUC5B*, *CDX2* – green; GEPIA top 12 genes: *CEACAM5*, *CEACAM6*, *CLDN3*, *CLDN4*, *CLDN7*, *REG4*, *MUC3A*, *MUC13*, *PI3*, *UBD*, *AOC1*, *CDH17* – black; Other: *TP53*, *APC*, *CDKN2A*, *FHIT* – red. **e**, The reference gastric tumour-normal dataset (Sathe et al.) for the LSI projection is shown with key cellular populations coloured according to their expression phenotype, as denoted in the legend. This panel is identical to that shown in Fig. 4c. **f–h**, Showing the LSI projection of individual cultures for donors 1, 2 and 3.

Phenotypic and transcriptional changes during in vitro evolution were evaluated based on growth dynamics of TP53^–/–^ HGO cultures and single-cell RNA sequencing (scRNA-seq) at early, mid and late time points (Fig. 3a, Extended Data Figs. 1 and 3a, Supplementary Table 2 and Methods). We investigated changes in cell proliferation by fitting a Loess regression model to cell numbers at each passage, using growth derivative and fold change as a surrogate for fitness. Higher growth derivatives were observed at late and mid versus early time points (Fig. 3b and Supplementary Fig. 15a); the use of raw cell numbers yielded similar results (*P* = 0.003, two-way repeated-measures analysis of variance; Supplementary Fig. 15b). scRNA-seq of 12 cultures (seven *TP53*^–/–^, two *APC*/*TP53* and three WT) yielded 31,606 cells for analysis following quality control (Fig. 3c, Extended Data Fig. 5a,b, Supplementary Fig. 16, Supplementary Table 6 and Methods). Normal gastric tissue markers were expressed in WT HGOs from the three donors, including pit mucosal cell (PMC) markers (*MUC5AC*, *TFF1*, *TFF2*, *GKN2*) in D1, enterocyte markers (*FABP1*, *FABP2*, *ANPEP*, *PHGR1*, *KRT20*) in D2 and gland mucosal cell (GMC) markers (*MUC6*, *PGC*, *TFF2*, *LYZ*) in D3, but were heterogeneous in *TP53*^–/–^ HGOs (Fig. 3c–e, Extended Data Fig. 5, Supplementary Fig. 16 and Supplementary Table 6).

The mucosal-like phenotype in WT cultures, defined by mucin and TFF gene expression, was lost following *TP53*^*–/–*^ in D1 and D3. Additionally, in D1 intestinal goblet cell-specific markers—including *TFF3*, *WFDC2* and *MUC5B*—were upregulated at the late time point, as commonly seen in intestinal metaplasia^36^. GC-associated genes, including claudins (*CLDN3*, *CLDN4*, *CLDN7*) and the carcinoembryonic antigen (CEA) family (*CEACAM5*, *CEACAM6*) increased in expression over time in D1 and D3. The inverse was observed in D2 cultures, plausibly due to an inflamed biopsy and the predominance of enterocytes in WT culture^14^ (Extended Data Fig. 5a,c). The absence of *MUC5AC* following *TP53*^*–/–*^ and increase in *CEACAM6* expression was verified by immunofluorescence staining in D3C2 (Supplementary Fig. 15c).

We investigated the overlap in transcriptional features across *TP53*^*–/–*^ HGOs by intersection of significantly differentially expressed genes (DEGs) from early to late time points across the six cultures with scRNA-seq data. In total, 13 consistently upregulated and 40 downregulated genes were identified (Bonferroni corrected *P* < 0.05, Wilcoxon rank-sum test; Fig. 3e and Supplementary Table 6). Upregulated genes included *CLDN4*, *TM4SF1* and *ZFAS1*, which are implicated in GC^22,37^, whereas those downregulated included *SPTBN1*, a cytoskeletal protein involved in TGFβ signalling^38^, and mucin production modulators *LYZ* and *TFF2*.

Last, we assessed pathway-level changes by gene set enrichment analysis (GSEA) of DEGs in both late versus early and mid versus early time points (Fig. 3f and Supplementary Table 6). Several pathways were enriched across multiple cultures and donors, including upregulation of tumour necrosis factor (TNF) signalling via nuclear factor kappa-light-chain-enhancer of activated B cells (NF-κB), as reported in CIN tumours^39^ and comparisons of GC versus normal tissue^40^ (four of six cultures), apoptosis (five of six cultures) and hypoxia (five of six cultures). Downregulated pathways included MYC, E2F targets and G2M checkpoints, although these were more variable and probably reflect survival programmes. Thus, despite heterogenous single-gene trajectories, pathways implicated in malignancy were shared across cultures and donors.

## Emergence of malignant expression states

**Fig. 4:**
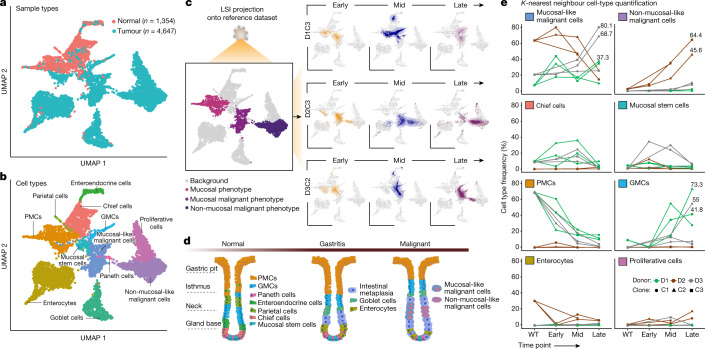
Unsupervised assessment indicates progression towards malignant transcriptional states. **a**,**b**, UMAP embedding of 6,001 epithelial cells from the Sathe et al. gastric tumour–normal scRNA-seq dataset^41^, coloured according to histology (**a**) and assigned cell type (**b**). Detected cell types included PMCs, GMCs, chief cells, parietal cells, enterocytes, enteroendocrine cells, goblet cells and proliferative cells, as well as two types of malignant cell (mucosal-like and non-mucosal-like). **c**, LSI projection of *TP53*^*–/–*^ HGOs sampled at early (orange), mid (blue) and late (purple) time points onto the reference dataset (left), coloured by cellular phenotypes of interest, providing orientation for the LSI projection of the three HGO cultures at the specified time points (right). The density of projected cells is highlighted using two-dimensional density distribution. LSI, latent semantic index. **d**, Schematic representation of shifts in cell populations proposed to accompany the transition from normal tissue to gastritis and that can lead to intestinal metaplasia and malignancy, adapted from ref. ^40^. **e**, Projected cell type frequencies based on the 25 nearest neighbours in HGOs over time. Panel **d** created with BioRender.com.

To identify pathologic features we projected HGO longitudinal scRNA-seq data onto a reference atlas comprising both normal and GC scRNA-seq^41^ (Methods). Restriction of the reference to epithelial cells yielded 6,001 cells (1,354 normal, 4,647 tumour) assigned to distinct cell type clusters using literature-derived marker genes^40–42^. Two tumour cell clusters emerged, comprising mucosal-like malignant and non-mucosal-like malignant cells^41^, the latter including malignant markers (*KRT17*, *KRT7*, *LY6D*) but lacking mucosal markers (*MUC5AC*, *TFF2*, *TFF1*) (Fig. 4a). PMCs, GMCs, chief cells, parietal cells, enterocytes, enteroendocrine cells, goblet cells and proliferative cells were also assigned to clusters (Fig. 4b).

We next projected batch-corrected scRNA-seq data from early, mid and late time points individually onto the reference embedding to identify gastric cell types that were most similar (Fig. 4c, Extended Data Fig. 5e–h and Methods). Because the reference atlas lacked preneoplastic populations, cells were projected onto either normal or tumour cell state in which the majority of HGO cells mapped onto the latter. Shifts in cell states over time were evident for all cultures, some of which have been implicated in the normal-to-gastritis transition that can lead to intestinal metaplasia and ultimately malignancy (shown schematically in Fig. 4d). Changes in cell type frequencies were quantified for each HGO culture by identification of the 25 nearest neighbours (NNs) in the reference population (Fig. 4e). An increase in mucosal-like malignant cells was observed in three of seven cultures at the late time point, with 68.7, 80.1 and 37.3% of NNs being mucosal-like malignant cells for D3C2, D3C3 and D1C1, respectively. By contrast, for D2, mucosal-like malignant cells decreased whereas non-mucosal-like malignant cells increased from WT to the late time point (D2C2, 45.6%; D2C3, 64.4%, NNs) (Fig. 4e), explaining transcriptional differences relative to D1 and D3 (Fig. 3). Notably, approximately 30% of cells in D2WT projected near enterocytes, potentially contributing to gastritis-like features and underlining the transcriptional similarity between enterocytes and malignant cells^42^. WT cultures from D1 and D3 exhibited predominantly mucosal phenotypes. The decrease in mucosal gene expression suggests that the evolved *TP53-*deficient HGOs were en route towards intestinal metaplasia and malignancy, albeit at different rates, corroborating the supervised analyses based on specific marker genes. Although our HGO cultures harbour hallmarks of CIN GC, they do not exhibit evidence of histologic transformation (Supplementary Fig. 15d).

## Deterministic growth of rare subclones

**Fig. 5:**
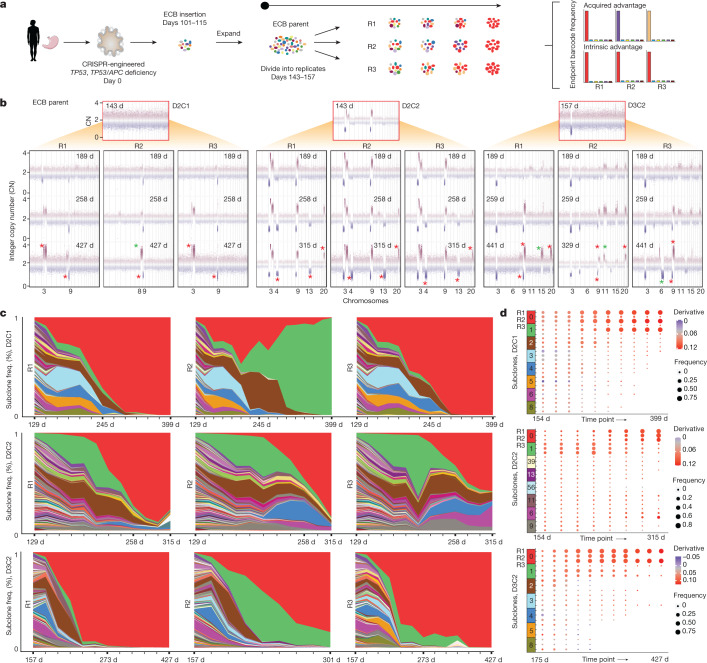
Lineage tracing showing subclonal dynamics and deterministic outgrowth. **a**, Overview of prospective lineage tracing studies in *TP53*^–/–^ HGOs using ECBs. The ECB parental population was split into replicates, and individual cultures evolved in parallel and subject to longitudinal barcode sequencing, showing subclonal dynamics and assessment of intrinsic or acquired fitness advantages amongst replicate cultures. **b**, CNA profiles were assessed by sWGS before the introduction of the ECB in the parental line and across replicate ECB cultures at multiple time points. Red asterisks denote CNAs present in at least two replicates but not in the parental population; green asterisks denote CNAs unique to one replicate. Only chromosomes harbouring newly arisen CNAs (not present in the parental population) are numbered, for simplicity. **c**, Muller plots depicting ECB frequencies (assessed by barcode sequencing) over time, where each colour represents a distinct subclone in each replicate. Note that, for D3C2, R2 (D3C2R2), the barcode was lost around day 273. **d**, Dot-plots indicating ECB subclone frequency (indicated by size) and estimated growth curve derivative per subclone (indicated by colour). Image of stomach in **a** is from Servier Medical Art, CC BY 3.0. Source Data

We next leveraged our HGO models to characterize preneoplastic subclonal dynamics at cellular resolution via prospective lineage tracing with high-complexity cellular barcodes. To jointly recover lineage and transcriptional states we developed expressed cellular barcodes (ECBs), which uniquely label each cell (Supplementary Fig. 17a,b and Methods). Five *TP53*^*–/–*^ (D1C1, D1C3, D2C1, D2C2, D2C3) and one *TP53*^*–/–*^, *APC*^*–/–*^ (D3C2) culture were transduced with ECB lentivirus between days 101 and 115 and evolved in parallel to the non-barcoded cultures for over 1 year. All cultures were Sanger sequenced at multiple time points to verify *TP53*/*APC* deletion clonality, resulting in the exclusion of D1C3 (Supplementary Fig. 17c,d). Each ECB parental line was split into three replicates to evaluate the reproducibility of clonal dynamics, in which outgrowth of the same subclone is assumed to reflect an intrinsic fitness advantage and divergent subclone dominance suggests acquired fitness differences (Fig. 5a).

**Extended Data Fig. 6:**
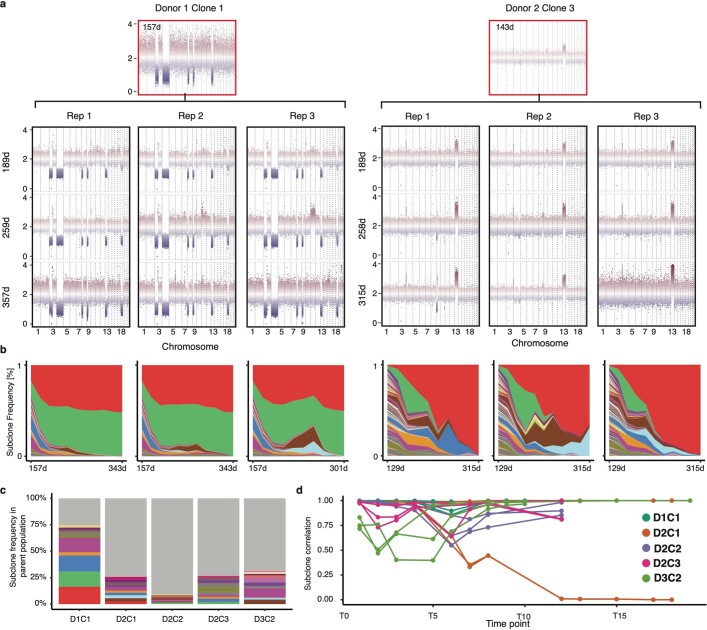
Subclone growth and frequency comparison and barcode trajectories for D1C1 and D2C3. **a**, sWGS of the parental population (top) and three time points for D1C1 and D2C3, replicates 1-3. **b**, Muller plots of the ECB subclone frequency over time for the cultures shown in panel a reveals similar lineage dynamics across replicates and deterministic outgrowth. **c**, Barplot depicting frequencies of the top 10 subclones in the parental population and the winning subclone (which was not one of the top subclones in the parental population). All other subclones coloured gray. **d**, Pairwise Pearson correlation over time between replicate cultures for D1C1, D2C1, D2C2, D2C3 and D3C2. Time points correspond to passage times, where intervals between passages are approximately two weeks. Source Data

Longitudinal sWGS of these long-term ECB cultures demonstrated marked reproducibility at the genomic level, with recurrent CNAs shared across replicate cultures (Fig. 5b, Extended Data Fig. 6 and Supplementary Fig. 18). For example, in D2C2 new CNAs emerged around day 258 (loss of chr4q and chr13, gain of chr20q) across all three replicates. In D2C1, R2 (D2C1R2) gain of chr8q was detected by day 258 and persisted (Fig. 5b) but was mutually exclusive with gains of chr3q in R1 and R3. By contrast, CNAs in different cultures from the same donor were more variable (Fig. 1c).

Through DNA sequencing of ECBs at regular intervals, we estimated the relative abundances of subclones over time and constructed Muller plots to visualize clonal dynamics (Fig. 5c). Colours were assigned to barcodes based on subclone frequencies across replicates within a culture, with the highest-frequency subclone coloured red. For example, the red band in D2C1R1 represents the same barcoded subclone as in D2C1R2 and D2C1R3. For each culture (except D2C1R2) the same (red) subclone became dominant across all replicates (Fig. 5c), consistent with an intrinsic fitness advantage and deterministic outgrowth (Fig. 5a). For D2C1 replicates R1 and R3 the red subclone became dominant in line with their shared CNA profiles whereas in R2 the green subclone, which acquired a chr8q gain (spanning the *MYC* oncogene), overtook the population. Intriguingly, brown and green clones expanded concomitantly before going extinct, suggesting their mutual dependence.

Of note, subclone frequency correlations over time across replicates was generally high, reflecting similar subclonal dynamics within a culture and similar patterns across cultures (Extended Data Fig. 6). Especially striking was the remergence of the blue and purple subclones in D2C2R2 and D2C2R3 at around 200 days (Fig. 5c). By construction of subclone-specific growth curves and estimation of their derivatives, we found that ‘winning’ subclones had high initial fitness and increased in proliferative capacity over time (Fig. 5d and Methods). Thus lineage tracing shows reproducible dynamics across replicate cultures, with adaptive lineages sweeping rapidly to fixation and dominant clones comprising 75% (median across cultures) of the population by day 144 post ECB transduction (Supplementary Table 7). These patterns are reminiscent of rapid adaptation in isogenic microbial populations attributable to standing variation in the initial population^43,44^.

## Molecular features of winning subclones

**Fig. 6:**
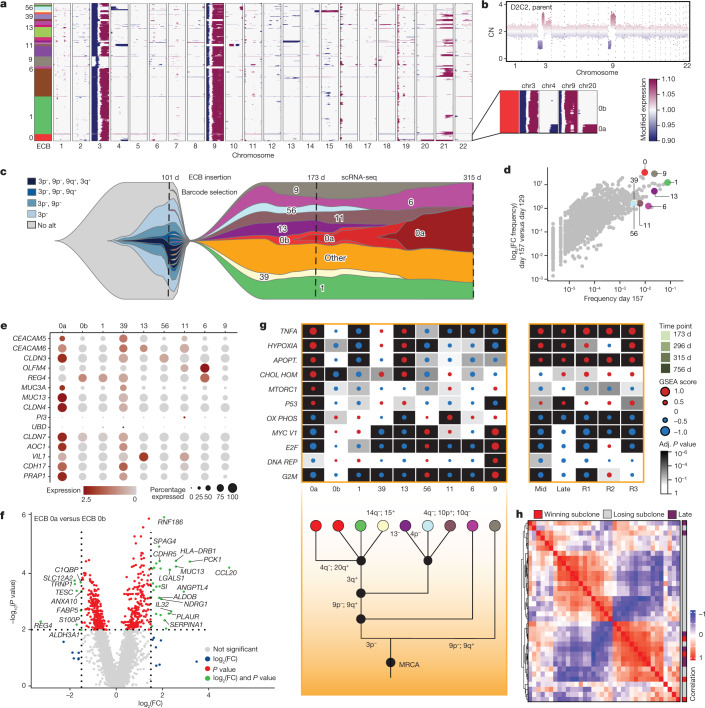
Genotype-to-phenotype mapping defines molecular determinants of winning subclones. **a**, Inferred CNA heatmap from scRNA-seq data for D2C2R2 at day 173, where each row represents a cell. Colour bar at the left indicates the ECB to which each cell maps. Numbered barcodes were selected for further investigation. Inset shows a subpopulation within ECB-0 with additional CNAs, termed 0a, and the ECB-0 parent subclone is termed 0b. **b**, CNA profile for the D2C2 parental population (also shown in Fig. 5b). **c**, Fishplot schematic illustrating the link between lineage (ECBs) and CNA subclones. To facilitate visualization, subclones of interest (denoted in **a**) are shown and the remainder grouped as ‘other’; all values are log transformed. **d**, Scatterplot comparing subclone frequency at day 157 and log_2_(FC) between days 129 and 157. All subclones are shown, with those of interest highlighted as in **a**. **e**, Dot-plot showing the expression of top DEGs based on GEPIA of gastric cancers. **f**, Volcano plot illustrating DEGs from comparison of the winning subclone 0a and its parental subclone 0b. Vertical and horizontal lines correspond to absolute log_2_(FC) values of 1.5 and *P* < 0.01 (two-sided Wilcoxon rank-sum test, not corrected for multiple testing), respectively. **g**, GSEA heatmap from MsigDB Hallmark gene sets showing the most significantly altered pathways (Kolmogorov–Smirnov statistic, Benjamini–Hochberg adjusted, two-sided) for specific subclones at day 173 (left) and later time points for the same culture (right). A manually reconstructed phylogeny is shown below. **h**, Pairwise Spearman correlation between samples based on GSEA score for the top ten most altered pathways for late relative to early time points and for subclones from multiple ECB replicate experiments. MRCA, most recent common ancestor. Source Data

**Extended Data Fig. 7:**
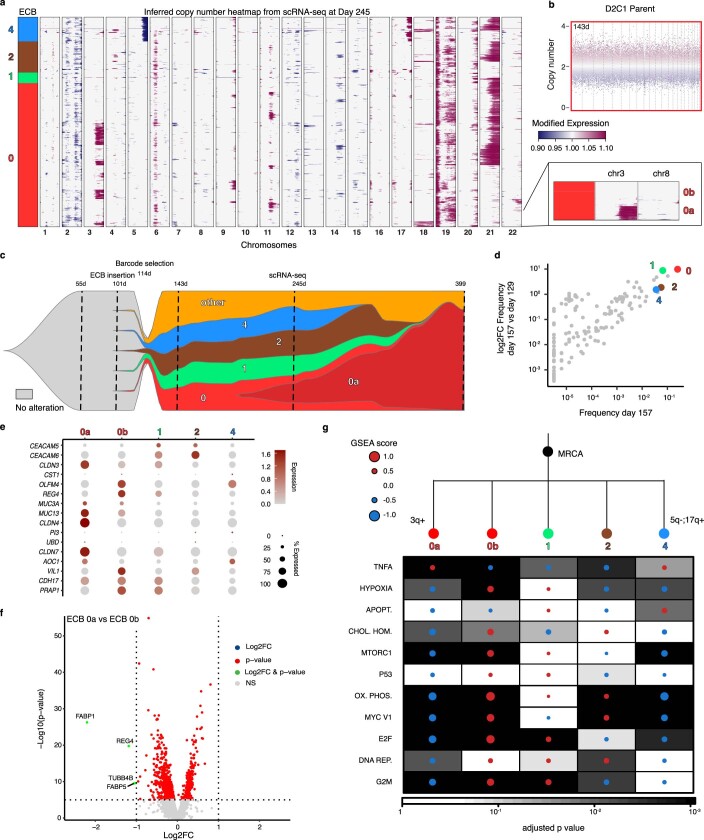
Linking single-cell genotypes to their transcriptional phenotypes in D2C1R1. **a**, Inferred copy number heatmap from scRNA-seq data at day 245, analogous to Fig. 6a. **b**, Copy number plot of parent population for D2C1. **c**, Fishplot schematic illustrating the link between lineage (ECBs) and copy number based subclones, similar to Fig. 6b. The actual subclone frequencies are shown in Fig. 5c. **d**, Scatterplot comparing subclone frequencies at day 157 and the fold change between days 129 and 157 for all subclones. Subclones of interest are highlighted as in panel a. **e**, Dotplot showing the expression of top differentially expressed genes (DEGs) based on GEPIA (gene expression profiling interactive analysis) of gastric cancer (GC). **f**, Volcano plot illustrating DEGs from the comparison of the winning subclone 0a and its parent subclone 0b. Vertical and horizontal lines correspond to absolute log2FC values of 1 and p-values < 0.00001 (Wilcoxon rank sum test, not corrected for multiple testing) respectively. **g**, GSEA heatmap from MSigDB Hallmark gene sets showing the most significantly altered pathways for the highlighted subclones (right; Kolmogorov-Smirnov statistic, Benjamini-Hochberg adjusted, two-sided). A manually reconstructed copy number phylogeny is shown above. This visualization is equivalent to Fig. 6f. Source Data

**Extended Data Fig. 8:**
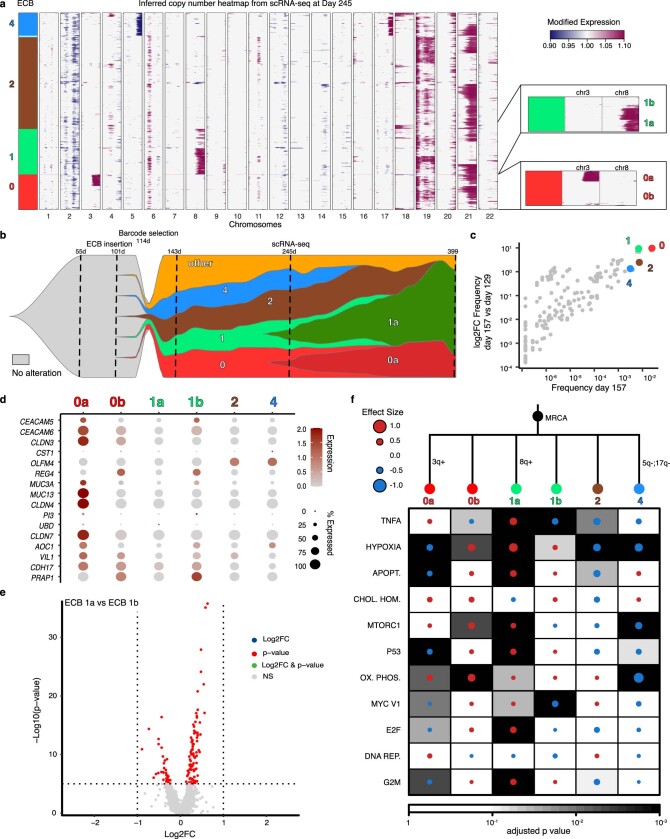
Linking single-cell genotypes to their transcriptional phenotypes in in D2C1R2. **a**, Inferred copy number heatmap from scRNA-seq data at day (d) 245, analogous to Fig. 6a. **b**, Fishplot schematic illustrating the link between lineage (ECBs) and copy number based subclones, similar to Fig. 6b. The actual subclone frequencies are shown in Fig. 5c. **c**, Scatterplot comparing subclone frequencies at day 157 and the fold change between days 129 and 157 for all subclones. Subclones of interest are highlighted as in panel a. **d**, Dotplot showing the expression of top differentially expressed genes (DEGs) based on GEPIA (gene expression profiling interactive analysis) of gastric cancer (GC). **e**, Volcano plot illustrating DEGs from the comparison of the winning subclone 0a and its parent subclone 0b. Vertical and horizontal lines correspond to absolute log2FC values of 1 and p-values < 0.00001 (Wilcoxon rank sum test, not corrected for multiple testing) respectively. **f**, GSEA heatmap from MSigDB Hallmark gene sets showing the most significantly altered pathways for the highlighted subclones (right; Kolmogorov-Smirnov statistic, Benjamini-Hochberg adjusted, two-sided). A manually reconstructed copy number phylogeny is shown above. This visualization is equivalent to Fig. 6f. Source Data

**Extended Data Fig. 9:**
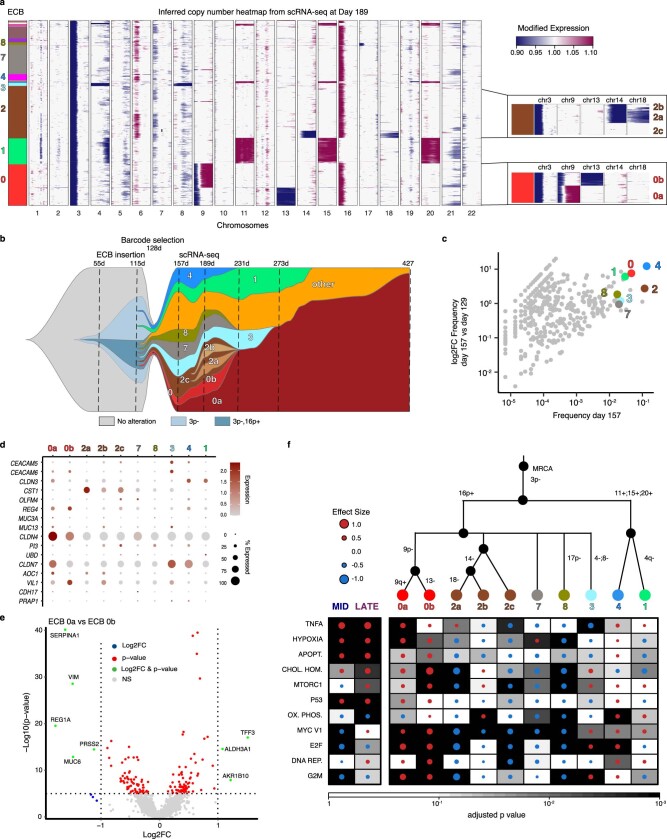
Linking single-cell genotypes to their transcriptional phenotypes in in D3C2R1. **a**, Inferred copy number heatmap from scRNA-seq data at day (d) 189, analogous to Fig. 6a. **b**, Fishplot schematic illustrating the link between lineage (ECBs) and copy number based subclones, similar to Fig. 6b. The actual subclone frequencies are shown in Fig. 5c. **c**, Scatterplot comparing subclone frequencies at day 157 and the fold change between days 129 and 157 for all subclones. Subclones of interest are highlighted as in panel A. **d**, Dotplot showing the expression of top differentially expressed genes (DEGs) based on GEPIA (gene expression profiling interactive analysis) of gastric cancer (GC). **e**, Volcano plot illustrating DEGs from the comparison of the winning subclone 0a and its parent subclone 0b. Vertical and horizontal lines correspond to absolute log2FC values of 1 and p-values < 0.00001 (Wilcoxon rank sum test, not corrected for multiple testing) respectively. **f**, GSEA heatmap from MSigDB Hallmark gene sets showing the most significantly altered pathways for the highlighted subclones (right; Kolmogorov-Smirnov statistic, Benjamini-Hochberg adjusted, two-sided). A manually reconstructed copy number phylogeny is shown above. The visualization is equivalent to Fig. 6f. Source Data

To investigate the targets of selection and how they change over time and across populations, we leveraged ECBs jointly capturing lineage and transcriptional states in individual cells. Specifically, we sought to characterize the molecular features of winning subclones that dominated the population after prolonged evolution by performing scRNA-seq for several donors and replicates at selected time points when the population was heterogeneous. For D2C2R2, which was sampled at day 173, 1,284 cells passed quality control and we identified 20 subclones with at least ten cells, all of which were among the top 38 most frequent ECBs based on barcode sequencing. Arm-level CNAs were inferred from the scRNA-seq data using *inferCNV* (Methods), showing numerous subclone-specific CNAs (Figs. 5b and 6a and Methods). Reassuringly, aggregate CNA landscapes were concordant with WGS data and scDNA-seq showed profiles and frequencies similar to subclone-specific CNAs inferred from scRNA-seq (Supplementary Fig. 19 and Methods). A detailed examination of this replicate (D2C2R2) showed complex evolutionary dynamics amongst coexisting subclones. Most cells comprising the winning subclone (ECB-0, red) acquired chromosome 3p^–^, 3q^+^, 9p^–^ and 9q^+^ alterations early because these events were clonal or nearly clonal in the parent population at day 143 (Fig. 6a,b). A subpopulation within ECB-0 (termed 0a) additionally acquired chr4q^–^ and chr20q^+^ and ultimately became dominant, with these alterations present in roughly 90% of the population at day 315 (Fig. 5b and Supplementary Fig. 20). Similar dynamics were seen across all replicate cultures in which winning subclones contained a nested CNA-defined subclone (Extended Data Figs. 7–9). These patterns may reflect a ‘rich-get-richer’ effect whereas fitness advantages acquired early drive clonal expansions, thereby increasing the likelihood of additional alterations that fuel growth^45^.

Because successful subclones consistently acquired additional genetic diversity, we sought to investigate the functional relevance of these events, focusing on a subset of subclones with divergent CNAs. As an example, D2C2R2 consisted of at least five different CNA clones at the time of barcode insertion (Fig. 6c and Supplementary Table 8). Multiple instances of convergent evolution were evident within this culture, in which subclones acquired the same CNA independently, implying stringent selection. For example, ECB-0a, ECB-11 and ECB-56 each lost variable-sized regions of chr4q. ECB-9 lacked common early alterations including chr3p^–^, but subsequently acquired chr9p/q alterations. Despite the incomplete set of CNAs, the growth of ECB-9 closely trailed that of the winning subclone (ECB-0; Fig. 6d). Convergent CNA evolution was also evident across cultures, in which chr15 and chr20 amplifications were present in the majority of cells in D3C2R1 at day 441, and these events plus chr11 amplification were present in R2 and R3 subclones by day 259.

**Extended Data Fig. 10:**
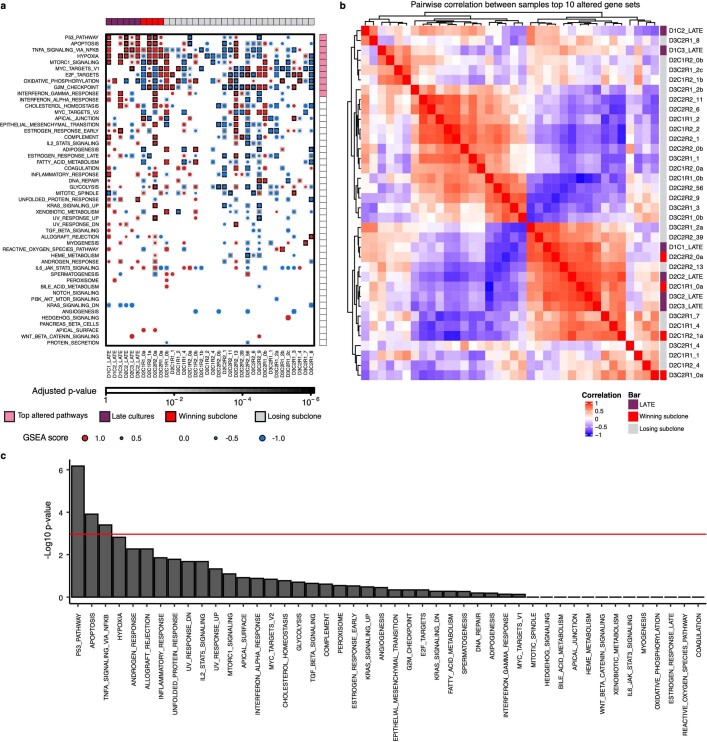
Enrichment of similar gene sets between winning subclones and *Late* cultures. **a**, GSEA heatmap from MSigDB Hallmark gene sets for each barcoded culture, including replicates: D2C2-R2 (day 173), D2C1R1 (day 245), D2C1-R2 (day 245), D3C2R1 (day 189), as well as non-barcoded *Late* cultures for D1C1, D1C2, D1C3, D2C2, D2C3 and D3C2. The GSEA score is indicated by dot size and coloured according to the directionality of expression profiles (up, red; down, blue). Background shading indicates statistical significance (Kolmogorov-Smirnov statistic, Benjamini-Hochberg adjusted, two-sided) **b**, Winning subclones and *Late* samples cluster together based on GSEA score. Pairwise spearman correlation between samples based on GSEA scores for the top 10 most altered pathways for all *Late* and winnings subclone samples. The most altered pathways are shown in a. This plot is identical to Fig. 6g but with sample annotations included. Note that the winning subclones (marked in red) cluster with the *Late* samples for D1C1, D2C2, D2C3 and D3C2 which exhibit a more malignant phenotype compared to *Late* samples D1C2 and D1C3 based on the LSI projection (Fig. 4c,d, Extended Data Fig. 5). **c**, Fisher exact test of independence (two-sided), comparing significance for each pathway, and status for each sample - *Late* and winning subclones (n = 16) relative to all other subclones analysed (n = 29). The red line indicates the significance threshold (0.05) with Bonferroni correction. Source Data

Although highly fit subclones differed in genomic landscapes, we reasoned that they would share transcriptional programmes. Indeed D2C2R2, the winning subclone 0a (but not its parent, 0b) exhibited high expression of several GC genes including *CEACAM5*, *CEACAM6*, *CLDN3*, *CLDN4* and *CLDN7* (Fig. 6e). These genes were also highly expressed in winning subclones of all other replicate cultures, except for ECB-1a (green) in D2C1R2, which acquired 8q gain (Extended Data Figs. 7e, 8d and 9d). The winning subclone, 0a (versus 0b), also upregulated GC genes, including *RNF186* which regulates intestinal homeostasis and is associated with ulcerative colitis^46^; *MUC13*, which encodes a transmembrane mucin glycoprotein^47^; *CCL20*, a chemokine and candidate biomarker^48^; and *LGALS1* (galectin-1), which promotes epithelial–mesenchymal transition, invasion and vascular mimicry^49^ (Fig. 6f and Supplementary Table 9). GSEA analysis, comparing the winning subclone in D2C2 with all other cells, showed upregulation of several pathways including TNF signalling via NF-κB, as well as hypoxia, apoptosis and p53 (Fig. 6g, Extended Data Fig. 10a and Supplementary Table 9). These same pathways were upregulated in three barcoded replicates for D2C2 at the final time point (day 315), as well as in the non-barcoded D2C2 culture at mid and late time points (relative to early) (Fig. 6f, right) and in other donors/cultures (D1C1, D1C2, D1C3, D2C3, D3C2) (Fig. 3f). Moreover, these pathways were upregulated in winning subclones from independent barcoded donors/cultures (Extended Data Figs. 7–9), including the divergent subclone (ECB-1a, green) in D2C1R2 (Fig. 5c and Extended Data Fig. 8f), emphasizing their reproducibility. More generally, strong concordance between winning subclone and non-barcoded late subclones was observed across the top ten altered gene sets irrespective of mycoplasma levels, antibiotic treatment and other sources of biological and technical variation (Supplementary Fig. 21 and Methods). Similarly, winning subclones clustered with late cultures, which exhibited malignant transcriptional states based on unsupervised LSI projection (D1C1, D2C2, D2C3 and D3C2) (Fig. 6h and Extended Data Fig. 10a,b). Notably, there was a significant difference in the activation of p53, apoptosis and TNF signalling via NF-κB pathways (Fisher’s exact test, Bonferroni corrected *P* < 0.05) between late (relative to early) and winning subclones compared with all other subclones (Extended Data Fig. 10c). These data highlight convergent phenotypic evolution in which the early activation of specific pathways is selectively advantageous, canalizing cells towards malignancy.

## Discussion

Through multiyear experimental evolution of *TP53*-deficient HGO cultures, we model preneoplastic evolution and genotype–phenotype relationships following this common initiating insult. Remarkably, *TP53* deficiency was sufficient to recapitulate multiple hallmarks of CIN GC including aneuploidy, specific CNAs, SVs and transcriptional programmes, emphasizing the importance of cell-intrinsic processes during premalignant evolution. Although aneuploidy propagates heterogenous evolution, our data show preferred orders in the acquisition of CNAs, with early loss of chr3p and 9p frequently followed by biallelic inactivation of *CDKN2A* and/or *FHIT* and relatively late gain of 20q. Such preferred mutational orders have been described during tumorigenesis, most notably in the colon, but the resolution of inferences from cross-sectional data or established tumours is inherently limited^12,50^. Evolutionary phases in which deletions preceded whole-genome doubling and subsequent amplifications were recently reported in a murine model of *KrasG12D*, *Trp53*-deficient pancreatic cancer, but neither gene nor chromosome level orderings were seen in this system^51^.

Our *TP53*^–/–^ HGOs exhibited transcriptional and genomic hallmarks of premalignant gastro-oesophageal lesions despite remaining histologically normal. This is consistent with the requirement for genomic perturbation for even the earliest stages of gastro-oesophageal carcinogenesis and the accrual of complex rearrangements years before cancer diagnosis^15,16,23^.

*TP53*^–/–^ HGOs appear to be on a trajectory similar to *TP53*-deficient Barrett’s oesophagus, for which the presumed cell of origin is gastric cardia^17^, and proposed biomarkers of progression to oesophageal cancer include CNA acquisition and SV burden^16,52^. These in vitro models thus recapitulate occult preneoplasia and mirror the latency of human tumorigenesis, with additional time or in vivo selective pressures evidently required for malignant transformation and further features of invasive disease such as whole-genome doubling or *ERBB2* amplification^22^.

The finding that *TP53* deficiency elicits a temporally defined order of genomic aberrations raises the possibility that these features may similarly predict progression to CIN GC. Future evaluation of this hypothesis will require annotated intestinal metaplasia tissue collection with long-term follow-up. Although *TP53* deficiency elicits tissue-specific alterations that may aid in the detection of high-risk lesions, this constrained evolutionary state is unlikely to persist indefinitely given ensuing genome instability, emphasizing the need for earlier detection.

By joint measurement of lineage, CNAs and transcriptional states in individual cells, we investigated the molecular basis of clonal expansions and fitness. This showed stringent selection and reproducible subclonal dynamics across replicate cultures in which the same, initially rare, subclone fixed in the population. Pervasive clonal interference was evident amongst subclones, accompanied by intermittent periods of relative stasis, suggesting that an optimal karyotype has yet to be achieved, as reported in colorectal adenoma^53^. Furthermore, we observed a marked degree of phenotypic convergence on common dominant pathways across cultures and donors, irrespective of mycoplasma infection and antibiotic treatment. This evolutionary reproducibility is particularly notable given these and other potential sources of technical and biological variation, and implies that any such effects are evidently modest relative to the overwhelmingly dominant effect of *TP53* inactivation.

These first in-kind measurements address open questions concerning selection and determinism in clonal evolution extendable to other tissues. In the vast space of initiating insults, recurrent tissue-specific alterations can be prioritized to identify selectively advantageous alterations, temporal order constraints and convergent phenotypes. Such constraints, due to epistasis, can show barriers to malignant transformation and potential therapeutic targets. We anticipate that our results will advance empirical and theoretical investigations of mutation, selection and genome instability in human cells, much as the long-term evolution experiments pioneered by Lenski and colleagues decades ago continue to yield fundamental insights into microbial adaptation^3,4^.

## Methods

A detailed description of the Materials and Methods is available in the Supplementary Information.

### Reagent availability

Requests for reagents should be directed to the corresponding author.

### Reporting summary

Further information on research design is available in the Nature Portfolio Reporting Summary linked to this article.

## Online content

Any methods, additional references, Nature Portfolio reporting summaries, source data, extended data, supplementary information, acknowledgements, peer review information; details of author contributions and competing interests; and statements of data and code availability are available at 10.1038/s41586-023-06102-8.

## Supplementary information

Supplementary Information This file contains Supplementary Figs. 1–21.

Reporting Summary

Supplementary Table 1 List of oligonucleotides utilized in this study.

Supplementary Table 2 Cell passaging information for all donors and cultures.

Supplementary Table 3 Summary of sWGS data including time points, coverage and quality control metrics and genomic alterations.

Supplementary Table 4 Summary of WGS data including time points, coverage and quality control and ploidy estimates.

Supplementary Table 5 CNAs accompanying fishplot schematics.

Supplementary Table 6 scRNA sequencing results including quality control metrics, top differentially expressed genes and GSEA results.

Supplementary Table 7 Timing of clonal sweeps based on ECB sequencing data.

Supplementary Table 8 Subclone frequencies in fishplot schematics.

Supplementary Table 9 scRNA sequencing information for ECB subclones, top differentially expressed genes and GSEA results.

## Data Availability

Metadata and cellranger outputs are available at Zenodo (10.5281/zenodo.6401895). Expressed cellular barcodes (ECB) sequencing data are available at bioProject ID (PRJNA838456). Genomic sequencing and scRNA-seq data are available at dbGAP under accession no. phs003249.v1. Source data are provided with this paper.

## Source data

Source Data Figs. 1–3 and 5–6.
Source Data Extended Data Figs. 2, 4 and 6–10.

## Code availability

The computational methods, procedures and analyses summarized above are implemented in custom R and python, and bash scripts are available via the Curtis Lab: https://github.com/cancersysbio/gastric_organoid_evolution.
