# Supplementary material for: Deterministic evolution and stringent selection during preneoplasia
Source: Nature. 2023 May 31;618(7964):383–93. doi: 10.1038/s41586-023-06102-8 (PMC10247377; doi:10.1038/s41586-023-06102-8)
Supplement: Supplementary file 2 — Reporting Summary [file 41586_2023_6102_MOESM2_ESM.pdf]

nature portfolio

Corresponding author(s): Christina Curtis

Last updated by author(s): Apr 5, 2023

Reporting Summary

Nature Portfolio wishes to improve the reproducibility of the work that we publish. This form provides structure for consistency and transparency in reporting. For further information on Nature Portfolio policies, see our Editorial Policies and the Editorial Policy Checklist.

Statistics

For all statistical analyses, confirm that the following items are present in the figure legend, table legend, main text, or Methods section.

n/a	Confirmed	
☐	☒	The exact sample size (n) for each experimental group/condition, given as a discrete number and unit of measurement
☐	☒	A statement on whether measurements were taken from distinct samples or whether the same sample was measured repeatedly
☐	☒	The statistical test(s) used AND whether they are one- or two-sided<br>*Only common tests should be described solely by name; describe more complex techniques in the Methods section.*
☐	☒	A description of all covariates tested
☐	☒	A description of any assumptions or corrections, such as tests of normality and adjustment for multiple comparisons
☐	☒	A full description of the statistical parameters including central tendency (e.g. means) or other basic estimates (e.g. regression coefficient) AND variation (e.g. standard deviation) or associated estimates of uncertainty (e.g. confidence intervals)
☐	☒	For null hypothesis testing, the test statistic (e.g. F, t, r) with confidence intervals, effect sizes, degrees of freedom and P value noted<br>*Give P values as exact values whenever suitable.*
☒	☐	For Bayesian analysis, information on the choice of priors and Markov chain Monte Carlo settings
☒	☐	For hierarchical and complex designs, identification of the appropriate level for tests and full reporting of outcomes
☒	☐	Estimates of effect sizes (e.g. Cohen's d, Pearson's r), indicating how they were calculated

Our web collection on statistics for biologists contains articles on many of the points above.

Software and code

Policy information about availability of computer code

Data collection	Software was not used for data collection
Data analysis	Open source and custom programs were used for data analysis as detailed in the Supplementary Information.

For manuscripts utilizing custom algorithms or software that are central to the research but not yet described in published literature, software must be made available to editors and reviewers. We strongly encourage code deposition in a community repository (e.g. GitHub). See the Nature Portfolio guidelines for submitting code & software for further information.

Data

Policy information about availability of data

All manuscripts must include a data availability statement. This statement should provide the following information, where applicable:

- Accession codes, unique identifiers, or web links for publicly available datasets
- A description of any restrictions on data availability
- For clinical datasets or third party data, please ensure that the statement adheres to our policy

Data availability:
Metadata and Seurat objects are available via Zenodo: https://doi.org/10.5281/zenodo.6401895
Barcode, WGS and scRNA-seq data are available under bioProject ID: PRJNA838456. Genomic sequencing data is available at dbGAP under accession ID: phs003249.v1.

Human research participants

Policy information about studies involving human research participants and Sex and Gender in Research.

Reporting on sex and gender	Sex was recorded for participants.
Population characteristics	Human subjects over the age of 18 undergoing sleeve gastrectomy for weight loss were included.
Recruitment	Participants undergoing surgical procedures were approached for study enrollment and provided informed consent.
Ethics oversight	Tissue collection was approved by the Stanford University IRB in accordance with relevant guidelines and regulations.

Note that full information on the approval of the study protocol must also be provided in the manuscript.

Field-specific reporting

Please select the one below that is the best fit for your research. If you are not sure, read the appropriate sections before making your selection.

☒ Life sciences ☐ Behavioural & social sciences ☐ Ecological, evolutionary & environmental sciences

For a reference copy of the document with all sections, see nature.com/documents/nr-reporting-summary-flat.pdf

Life sciences study design

All studies must disclose on these points even when the disclosure is negative.

Sample size	For each of the three tissue donors, three independent genome-edited, single-cell derived cultures were established, corresponding to 9 lines. For barcoding studies, 5 independent lines were split into three technical replicates and passaged concurrently with one another (n=15), and along side the non-barcoded lines (n=9). Samples sizes are as noted for published genomic datasets.
Data exclusions	No data was excluded
Replication	Three independent single-cell derived genome edited cultures were generated for each donor specimen. Additionally, for 5 of these cultures, cellular barcoding studies were performed in 3 technical replicates each (n=15) and these lines were passaged in parallel to the parental (non-barcoded line).
Randomization	NA
Blinding	NA

Reporting for specific materials, systems and methods

We require information from authors about some types of materials, experimental systems and methods used in many studies. Here, indicate whether each material, system or method listed is relevant to your study. If you are not sure if a list item applies to your research, read the appropriate section before selecting a response.

Materials & experimental systems

n/a	Involved in the study	
☐	☒	Antibodies
☐	☒	Eukaryotic cell lines
☒	☐	Palaeontology and archaeology
☒	☐	Animals and other organisms
☒	☐	Clinical data
☒	☐	Dual use research of concern

Methods

n/a	Involved in the study	
☒	☐	ChIP-seq
☐	☒	Flow cytometry
☒	☐	MRI-based neuroimaging

Antibodies

Antibodies used	MUC2 GeneTex #GTX100664<br>MUC5AC Cell Signaling Technology #61193

	CEACAM6 Cell Signaling Technology #85102
Validation	Validated commercially available antibodies were used.

Eukaryotic cell lines

Policy information about cell lines and Sex and Gender in Research

Cell line source(s)	Clinical samples (gastric corpus tissue) were obtained with informed consent from three patients undergoing sleeve gastrectomy under an IRB approved protocol (# 11977) through the Stanford University Hospital Tissue Procurement Shared Resource facility. Specimens were confirmed to be non-malignant and were used to generate wild-type (WT) gastric organoid (GO) cultures and subsequently CRISPR/Cas9 edited cultures based on an established protocol, as detailed in the Methods.
Authentication	Clonal status of CRISPR-edited sites was verified via Sanger sequencing and confirmed by WGS at multiple time points (Methods).
Mycoplasma contamination	During the experiment, mycoplasma was detected and an antibiotic (normocin) was used to eliminate infections (see Supplemental Information, Methods: Evaluation of mycoplasma levels and the association with molecular features). We performed standard mycoplasma PCR testing and assessed mycoplasma levels across the experimental time course by mapping WGS data from all samples to multiple mycoplasma species and computed the reads per million. Replicate experiments were performed in mycoplasma-free conditions and demonstrate that mycoplasma infection was not associated with copy number aberrations or other molecular features.
Commonly misidentified lines (See ICLAC register)	NA

Flow Cytometry

Plots

Confirm that:

☒ The axis labels state the marker and fluorochrome used (e.g. CD4-FITC).

☒ The axis scales are clearly visible. Include numbers along axes only for bottom left plot of group (a 'group' is an analysis of identical markers).

☒ All plots are contour plots with outliers or pseudocolor plots.

☐ A numerical value for number of cells or percentage (with statistics) is provided.

Methodology

Sample preparation	Gastric organoids were stained with DAPI as single cell suspension
Instrument	Aurora Flow Cytometry System (Cytek Biosciences)
Software	Samples were analyzed with FlowJo
Cell population abundance	NA
Gating strategy	The gating strategy was chosen to identify live single cells prior to measuring DAPI content

☒ Tick this box to confirm that a figure exemplifying the gating strategy is provided in the Supplementary Information.
